# Does Motor Simulation Theory Explain the Cognitive Mechanisms Underlying Motor Imagery? A Critical Review

**DOI:** 10.3389/fnhum.2017.00072

**Published:** 2017-02-17

**Authors:** Helen O’Shea, Aidan Moran

**Affiliations:** School of Psychology, University College DublinDublin, Ireland

**Keywords:** simulation theory, motor imagery, motor cognition, functional equivalence, embodied cognition, emulation

## Abstract

Motor simulation theory (MST; Jeannerod, 2001) purports to explain how various action-related cognitive states relate to actual motor execution. Specifically, it proposes that motor imagery (MI; imagining an action without executing the movements involved) shares certain mental representations and mechanisms with action execution, and hence, activates similar neural pathways to those elicited during the latter process. Furthermore, MST postulates that MI works by rehearsing neural motor systems off-line via a hypothetical simulation process. In this paper, we review evidence cited in support of MST and evaluate its efficacy in understanding the cognitive mechanisms underlying MI. In doing so, we delineate the precise postulates of simulation theory and clarify relevant terminology. Based on our cognitive-level analysis, we argue firstly that the psychological mechanisms underlying MI are poorly understood and require additional conceptual and empirical analysis. In addition, we identify a number of potentially fruitful lines of inquiry for future investigators of MST and MI.

## Introduction

Motor simulation theory (MST; hereafter, simulation theory; Jeannerod, 1994, 2001, 2006a) offers a seminal explanation for how various action-related cognitive states such as “motor imagery” (MI; the mental rehearsal of actions without engaging in the movements involved; Moran et al., 2012), action intention (the translation of a desired movement into behavior; Haggard, 2005) and observation, are related to actual motor execution (ME) states. The cornerstone of MST is the idea that cognitive motor states activate motor systems in the brain that are similar to those triggered during actual action (Jeannerod, 2001, 2004, 2006a). Further, these motor systems can be rehearsed off-line via a putative simulation mechanism which allows the mind to anticipate action viability and potential action outcomes (Jeannerod, 2001). Similar neural activation during motor cognition and ME is assumed to occur because both states share motor representations in the mind – the idea that actions are internally (or mentally) generated according to a specific goal and in the absence of external environmental cues (i.e., the theory of action representation, see Jeannerod, 1994, 2004, 2006b; Pearson and Kosslyn, 2015). Specifically, MI and ME “are both assigned to the same motor representation vehicle” (Jeannerod, 1994, p. 190) with the representation being the “covert counterpart of any goal-directed action, executed or not” (Jeannerod, 2006a, p. 165). This correspondence between ‘simulated’ and executed action led to the ‘functional equivalence’ hypothesis (Jeannerod, 1994, 2001, 2006a), which maintains that “motor imagery … should involve, in the subject’s motor brain, neural mechanisms similar to those operating during the real action” (Jeannerod, 2001, pp. S103–S104).

At first glance, MST proposes a fertile hypothetical mechanism (“simulation”) that offers intriguing insights into motor cognition (e.g., see Grush, 2004; Gentsch et al., 2016) and the neurocomputational parallels between imagination and action (e.g., Conson et al., 2009; Case et al., 2015). On closer inspection, however, MST is hampered by problems arising from the inadequate specification of key postulates. For example, Jeannerod (2006a) claimed that “represented actions should involve a simulation of the mechanisms that normally participate in the various stages of action generation, including motor execution” (p. 130). However, these potential mechanisms are rarely delineated and when they are (e.g., see the schematic diagrams of action representations and associated texts, Jeannerod, 2004, 2006a), their precise operational details are rather vague.

In view of this problem of unclear mechanisms, the purpose of the present paper is twofold. Firstly, we aim to specify the main postulates of MST and to evaluate the evidence available to support them. Secondly, and more specifically, we wish to examine the adequacy of MST in explaining the cognitive mechanisms that underlie MI. In order to achieve these two objectives, the paper is organized as follows. To begin with, we shall explore Jeannerod’s understanding of simulation. This task requires analysis of different usages of the term simulation and a brief evaluation of alternative theories of MI. Following this, we present key postulates of MST and evaluate the evidence that supports them – although space restrictions preclude an in-depth analysis of the substantial research literature in question here. The next section will focus on the implications of simulation theory for understanding the cognitive mechanisms underlying MI. Here, we will consider the extent to which simulation theory is sufficient for explaining MI, and, if not, whether we should turn to other relevant conceptual approaches, such as emulation and grounded theories. We shall end by briefly sketching some aspects of MST that require further research. Although our paper builds on previous reviews of simulation-related topics (e.g., see Munzert et al., 2009; Pezzulo et al., 2013; Ridderinkhof and Brass, 2015), it differs from them in its critical evaluation of key postulates of MST and also in its consideration of possible cognitive mechanisms underlying MI.

## Simulation, Emulation, and Embodied Theories of MI

The term simulation has many referents in social and motor cognition domains. As the former have been discussed elsewhere (e.g., Shanton and Goldman, 2010; Gallagher, 2015), they fall outside the scope of our review. However, within the motor cognition domain, simulation was postulated by Jeannerod (2006a) to mean “the offline rehearsal of neural networks” (p. 129), and “activation of the motor system is a prerequisite for the simulation theory” (Jeannerod, 2001, p. S104). Other researchers, however, use the term with different emphasis. For example, simulation as conceptualized by grounded theories, is multimodal (not just motoric) and operates to achieve particular conceptual knowledge. Specifically, it is the re-enactment of previously experienced modal states (i.e., perceptual, motor, introspective, and proprioceptive states), which are held in memory as a multimodal representation (e.g., arising from stroking a dog; Barsalou, 2008), by using multiple concurrent modal simulations to acquire specific conceptual knowledge (e.g., concept of dog; Barsalou, 1999, 2008; Decety and Grèzes, 2006). Alternatively, the term simulation is used in terms of anticipatory-associative mechanisms rather than internal models or representations, and denote the idea that when an action is simulated it activates the same neural motor systems as it would during actual action, which then activate associative mechanisms facilitating sensory simulation (Hesslow, 2002, 2012). Anticipatory mechanisms are key to the simulation process, in that, the anticipated sensory consequences that arise from the preparatory stages of movement (including global goal stages) are associated with action simulation that prompts the next action … and so on (Hesslow, 2002). Thus, anticipatory and association processes ensure that continuous simulation occurs. Although both grounded (e.g., Barsalou, 2008) and association (Hesslow, 2002, 2012) simulation theories have not as yet been directly related to MI processes, each can broadly account for MI experience. Specifically, grounded and association simulation accounts contend that motor simulation can occur in the absence of external input, relying on the re-enactment of previously experienced events which are stored in multi-modal representational format (grounded theories) or previously reinforced associations (association simulation).

Let us now move from the meaning of simulation to the attempt to explain MI. Early theories of MI focused on explaining it in terms of the action-enhancement phenomenon of “mental practice” (MP; or the “cognitive rehearsal of a task in the absence of overt physical movement”; Driskell et al., 1994, p. 481; see also Di Rienzo et al., 2016). They posited that MI results in a faint innervation of key muscles involved in action execution which improves their strength, velocity, or control (psychoneuromuscular accounts; see Driskell et al., 1994; Guillot et al., 2007); or in the strengthening of motor representations through the cognitive organization of movement-element coding and binding (symbolic accounts; Driskell et al., 1994; Schack, 2004). Modern explanations of how MI works are more mechanistic, however, and largely centre on the theoretical concept of “forward modeling” in motor control theory (see, e.g., Wolpert et al., 1995; Wolpert and Flanagan, 2001) or embodied/grounded cognition (e.g., Wilson, 2002; Barsalou, 2008; Borghi and Cimatti, 2010). Regarding the latter, evidence exists that the body is (subliminally) involved during off-line motor cognitive states such as MI, thereby giving simulation an embodied dimension (e.g., through physiological activity, see, e.g., Collet and Guillot, 2010; Stinear, 2010). However, while motor images have sometimes been referred to as embodied mental states (Jeannerod, 2006a), there are more formal ideas of what embodiment is (see Wilson, 2002; Borghi and Cimatti, 2010). Typically, embodiment theories maintain that cognition does not exclusively rely on internal representations (or, at the extreme end, considers whether they even exist), as they place at their core the body’s interaction with the world and how this shapes the mind (Wilson, 2002). Thus, these theories claim that cognition, knowledge, and sensorimotor experience and information are tightly interconnected (Holt and Beilock, 2006). MST conceptualizes MI as a simulation of the covert or representational stage of the same executed action, and in this regard, appears incompatible with embodied cognition. To our knowledge, embodiment theories have yet to conceptualize MI within the framework.

Regarding the forward modeling of motor control theories, during action execution, temporal delays in sensory feedback interfere with the motor system’s ability to accurately control movement trajectory and kinematics (Wolpert and Ghahramani, 2000). To compensate for such delays, the consequences of an action are believed to be predicted, using an internal forward model of the motor-to-sensory transformation (i.e., efference copy of the motor command), which facilitates the formulation of sequential motor commands in the absence of actual sensory feedback (e.g., Wolpert and Ghahramani, 2000). Drawing on the concept of predictive forward modeling, emulation theory posits a specific *type* of simulation – a predictive process operating during MI that goes beyond MST’s simulation of efferent motor centres (motor-to-sensory transformation; Grush, 2004; Moulton and Kosslyn, 2009). Simulation according to MST rehearses the motor system and is guided exclusively by internal motor representations, whereas in emulation theory, motor *and* sensory systems are emulated in parallel (Grush, 2004). According to emulation theory, during MI, the efference copy of the motor command (forward model) drives body/environment emulators (i.e., motor and sensory representations) in order to simulate movement, proprioception, and kinesthesis. Accordingly, processes that are assumed to beneficially operate as adaptive motor control mechanisms during action execution are exploited in action imagination (Grush, 2004). Emulation theory posits that action execution (via efferent motor centres, albeit inhibited) and predicted action consequences are not sufficient for MI to arise. Instead, as MI is considered to involve a motor plan and proprioceptive and kinesthetic feelings, emulation specifies the need to also simulate the afferent sensory systems (thereby predicting sensory feedback). We will further discuss emulation theory after we discuss the postulates of, and evidence for, MST. It is important to note that all of the aforementioned accounts of simulation and current theories of MI highlight the interconnectedness between cognition and sensorimotor experience and information.

## Postulates of Motor Simulation Theory

Surprisingly, for such a widely cited theory, the key postulates of MST have not been summarized in one single publication to date. To fill this gap, the three central tenets of simulation theory are summarized below along with the evidence available to support them. As research employing a simulation theoretical framework is extensive, however, we will not engage in an in-depth analysis of this literature. Instead, we merely highlight salient findings that contribute to the discussion. Further, as a specific focus of the review is to understand a MST account of *MI*, this section will draw on literature referring specifically to this particular cognitive motor state (rather than to those of related states such as action observation; see Eaves et al., 2016).

A fundamental assumption of simulation theory is that action is preceded by the activation of a mental representation which contains most aspects of the future action. Thus, the mental representation of the motor action and that of the actual action form a continuum whereby the represented movement unfolds over time before culminating in eventual execution (Jeannerod, 1994, 2001, 2004; see **Table 1** for postulates). Accordingly, MST proposes that action information is represented and processed centrally and includes most of the content of an executed action, that is, its goal, plan, motor program and consequences. MST further claims that represented actions (i.e., all covert actions, e.g., MI) can function off-line (i.e., cognitively) by using the same mechanisms as actual action – except that execution is inhibited. This is possible as the motor system is part of cognitive network (Jeannerod, 1994, 1997, 2001, 2006a). Such a claim implies, firstly, that the motor system in the brain is involved during motor cognition, secondly, that neural activity observed during executed action is similar to that observed during represented action, thirdly, a functional equivalence between cognitive motor states, such as MI, and actual actions (i.e., both have a causal role in generating action), and finally, that inhibitory mechanisms operate to prevent overt movement during action representation.

**Table 1:** Key postulates of simulation theory.

(1)	Real action comprises a covert (representational) – overt (execution) continuum, where the covert/representational stage contains most aspects of the future action, that is, the goal, motor plan/program, and its consequences (Jeannerod, 1994, 1999, 2001, 2004, 2006a).
(2)	Action representations can operate off-line, via a simulation mechanism, as the motor system is part of a cognitive network (Jeannerod, 2001, 2006a).
3)	Represented (i.e., covert) actions rely on the same set of mechanisms as the real action they simulate, except that execution is inhibited (Jeannerod, 2004, 2006a).

## Evidence Relating to the Claims of Motor Simulation Theory

Several lines of investigation have been employed to explore the claims of simulation theory. These empirical strands include neurophysiological studies mapping brain activity during cognitive motor states and ME, neuropsychological and physiological studies exploring correlations between the different motor states, the effects of MI on subsequent motor performance, and empirical studies (largely employing mental chronometry paradigms). Compelling evidence that action representations exist as purely mental states that reside in and activate neural motor systems offline comes from neurophysiological and physiological investigations of the correspondences between actual and imagined actions (see, e.g., Collet et al., 2011; Hétu et al., 2013). Firstly, peripheral physiological measures, such as skin resistance, cardiovascular and respiratory rates, eye blinking activity and electromyography (EMG) activity have been found to correlate between actual and imagined movements (e.g., Boschker, 2001; Guillot and Collet, 2005a; Guillot et al., 2007; Papadelis et al., 2007; Collet et al., 2011). For example, during MI, cardiovascular and respiratory rates have been shown to increase proportionately with escalations in running speed on a treadmill (Decety et al., 1991). As physiological measures reflect autonomic activation which is largely outside voluntary control, their presence during MI indicates that they have a central origin (i.e., representational), and their similarity between ME and MI suggests that both movement types have comparable representational content (Jeannerod, 2006a).

Recent neuroimaging and analytical (e.g., functional connectivity methods; for review, see Westlake and Nagarajan, 2011) advances facilitate neural-level investigation that produces nuanced and sophisticated data regarding the neural organization underlying actual and imagined (i.e., represented) actions. **Table 2** summarizes the key anatomical structures typically involved during MI, in relation to those involved during ME (for overview of neuroanatomical regions generally associated with action execution, see, e.g., Tanji and Hoshi, 2008; Gallivan and Culham, 2015). As indicated in the table, research demonstrates that MI and ME share brain regions, namely, the PFC (including anterior cingulate cortex), pre-motor cortex (PMC), SMA, posterior parietal cortex (PPC), primary motor cortex (M1; although not consistently, see e.g., Lotze and Halsband, 2006; Munzert et al., 2009), cerebellum and the basal ganglia (see Jeannerod, 1995, 2001; Lotze et al., 1999; Grèzes and Decety, 2001; Munzert et al., 2009; Hétu et al., 2013; Lotze, 2013; Jiang et al., 2015; Ridderinkhof and Brass, 2015; Zhang et al., 2016). Further, studies exploring the characteristics of the motor system during brain resting state (RS), MI, and ME reveal a functional connectivity between PMC, SMA, and M1 during RS and ME in healthy participants (Bajaj et al., 2014) and during MI, ME, and RS in stroke patients (Bajaj et al., 2015). Interestingly, higher functional connectivity between prefrontal-parietal regions at RS results in greater activation in those areas during an imagined motor task (Saiote et al., 2016). Common active brain regions provide support for the claim that the motor system is part of a cognitive network, it is involved during MI, and that this involvement closely reflects that of ME. However, it should be noted that while evidence of common active brain regions during MI and ME supports the claims of MST, it also provides support for alternative theoretical explanations of MI, such as emulation or grounded simulations (Grush, 2004; Barsalou, 2008). The latter two similarly conceptualize simulation as being contingent on activity in the motor system – for the emulation or re-enactment (grounded) of motor states.

**Table 2:** Key anatomical brain regions associated with MI and Motor Execution (ME).

Anatomical region	MI	ME	Selected reference
dlPFC	Active	Active	Gerardin et al., 2000; Hanakawa et al., 2003; Kuhtz-Buschbeck et al., 2003; Hétu et al., 2013
SMA	Greater activity in rostral SMA Active pre-SMA	Active SMA No activity in pre-SMA	Stephan et al., 1995; Gerardin et al., 2000; Kuhtz-Buschbeck et al., 2003; Vry et al., 2012
PMC	Active	Active	Gerardin et al., 2000; Hanakawa et al., 2003; Munzert et al., 2009
Par	Greater activity in PPC and IPL	Greater activity in S1	Gerardin et al., 2000; Hanakawa et al., 2003; Hétu et al., 2013; Saiote et al., 2016
M1	Weak activation	Active	Munzert et al., 2009; Sharma and Baron, 2013; Bajaj et al., 2015; Saiote et al., 2016
Cerebellum	Greater activity caudally	Greater activity rostrally	Hanakawa et al., 2003; Ridderinkhof and Brass, 2015
Basal Ganglia	Active rostral caudate nucleus	Active caudal putamen	Gerardin et al., 2000; Hétu et al., 2013

*Par, parietal cortex; IPL, inferior parietal lobule; PPC, posterior parietal cortex; dlPFC, dorsolateral prefrontal cortex; PMC, premotor cortex; SMA, supplementary motor cortex; S1, primary somatosensory cortex; M1, primary motor cortex; d, dorsal; l, lateral.*

Studies examining the distributed nature of the neural networks involved during ME and MI have identified subtle differences in their underlying motor pathways. A shift in neural activity has been demonstrated in the parietal cortex from higher volumes at anterior loci (primary sensory area; S1) during actual action, to higher volumes at more posterior loci during MI (particularly IPL; Gerardin et al., 2000; Hétu et al., 2013; see **Table 2**). Increased activation in the PPC during MI, in relation to ME, reflects its pure representational quality, as this region, particularly the left IPL, is associated with the generation and storage of motor representations (Andersen and Buneo, 2002; Creem-Regehr, 2009; Gallivan and Culham, 2015). Further, regarding the SMA, activity during MI is greatest in the pre-SMA (rostral) whereas during ME, activity is more confined to the SMA proper (more caudal; e.g., Stephan et al., 1995; Gerardin et al., 2000; Kuhtz-Buschbeck et al., 2003; Wang et al., 2010). The pre-SMA projects to the dlPFC and is associated with higher-order cognitive motor processes, such as conscious action intention, motor control, selection, sequencing, and preparation (Cunnington et al., 1996; Lehéricy et al., 2004; Lau et al., 2006; Nachev et al., 2008; Moore et al., 2010). The fact that the dlPFC (Stephan et al., 1995; Gerardin et al., 2000; Hanakawa et al., 2003; Kuhtz-Buschbeck et al., 2003; Hétu et al., 2013) and the inferior frontal gyrus (IFG; see Jeannerod, 2006a) typically display greater activity during MI than during ME, highlights a distinction between the two action states, and challenges the claim that represented actions rely on the same mechanisms as the real action they simulate. Further, research demonstrates that while MI and ME activate dorsal networks connecting frontal-parietal regions, MI further activates ventral networks linking prefrontal-parietal regions (Vry et al., 2012). Distinct activity may reflect a greater requirement during MI for attentive awareness for maintaining information in mind (dlPFC) and inhibition of overt movement (IFG; Frith and Dolan, 1996; Pochon et al., 2001; Aron et al., 2003).

It is also worth noting that a trend of weaker neural activity during MI than during ME is generally reported in neuroimaging and effective connectivity studies (e.g., Lotze et al., 1999; Jeannerod, 2001; Stippich et al., 2002; Dechent et al., 2004; Solodkin et al., 2004; Gao et al., 2011; Sharma and Baron, 2013; Avanzino et al., 2015). This trend may reflect concurrent inhibitory processes which prevent action execution during MI, which would be consistent with the postulate of MST that ME and MI share common representations and activate similar neural motor systems, only movement execution is inhibited during MI (Jeannerod, 2001, 2006a).

If represented actions rely on the same mechanisms as ME then it is expected that damage to part of the motor system would similarly impair or prevent both action states (Jeannerod, 2001). Neuropsychological evidence demonstrates that the fronto-parietal network must be intact to perform accurate and effective MI (e.g., Schwoebel et al., 2002; McInnes et al., 2016; Oostra et al., 2016), and ME (e.g., Sirigu et al., 2004; Nachev et al., 2008). Further, interruption to the IPL (via transcranial magnetic stimulation, TMS, or injury) significantly reduces or destroys the ability to generate accurate motor images (Sirigu et al., 1996; Gerardin et al., 2000; Guillot et al., 2008; Buch et al., 2012; Lebon et al., 2012; McInnes et al., 2016; Saiote et al., 2016), acquire MI-based skill (Kraeutner et al., 2016), modify ineffective actual actions (Sirigu et al., 2004), or coordinate actual movements (see McInnes et al., 2016). It seems therefore, that the PPC is crucial for effective MI and ME, with both behaviors relying on the region’s functional properties, most likely for action intention processes (Sirigu et al., 2004; Tunik et al., 2007) and the generation of motor representations (Rushworth et al., 2003).

From the analysis above, it is apparent that a vast quantity of neural-level research within the simulation theoretical framework has contributed to identification of key neural circuitry underlying MI. Interpreting this evidence in general terms, we cautiously conclude that although MI and ME processes share certain brain *regions* relating to the motor system, the specific motor *networks* underlying these motor behaviors are not identical. Subtle but important differences exist, and these differences may relate to processes that are necessary and specific to each movement type/behavior. However, as the precise cognitive processes supporting MI have not, as yet, been delineated, any region – process/function associations in the context of MI are tentative.

Additional insight into the validity of the claims of MST may be gained from behavioral-level research, and in particular that employing mental chronometry paradigms (for reviews, see e.g., Guillot and Collet, 2005b; Guillot et al., 2012a; Moran and Toner, 2017). These paradigms assume that temporal indices (i.e., the time-course of information-processing behavior) can elucidate the underlying cognitive representations and mechanisms of behavior (Posner, 1978). Studies employing the mental chronometry paradigm demonstrate similar performance durations for actual and imagined actions during automatic (Decety and Michel, 1989; Maruff and Velakoulis, 2000; Papaxanthis et al., 2002) and cyclical (e.g., pedalo rowing, Munzert, 2002) movements, and during movements governed by physical laws such as Fitts’ law (i.e., speed-accuracy trade off, Decety and Jeannerod, 1996; Cerritelli et al., 2000). Congruence between the timing of actual and imagined movements suggests that the representational content, including motor rules (e.g., Fitts’ Law), and information-processing mechanisms underpinning actual action and motor representational states are similar.

In passing, a note of caution about mental chronometry paradigms in MI research is warranted. Specifically, they provide data only on the similarity of information processing occurring *overall* during ME and MI, and do not offer insight into the processing specifics involved. For example, mental chronometry methods offer information about the timing of mental processing but no information regarding the accuracy of MI processes (e.g., Guillot and Collet, 2005a). Currently, other than duration, we do not know exactly what mental chronometry actually measures during MI. This matter is significant considering that temporal inconsistencies between ME and MI have also been demonstrated in several contexts (see Guillot and Collet, 2005b; Guillot et al., 2012a), specifically, during complex tasks (Decety et al., 1989; Calmels et al., 2006; O’Shea and Moran, 2016), bimanual tasks (Dahm and Rieger, 2016), and tasks that involve added mass (Decety et al., 1989; Cerritelli et al., 2000). Further, expertise appears to be a factor in observations of temporal congruence, with experts achieving greater correspondence between actual and imagined movement performance times than novices (Reed, 2002). However, expert performers in music have also been found to exhibit temporal incongruence when a complex, bimanual task is involved (O’Shea and Moran, 2016). Thus, differences in actual and imagined performance durations may reflect distinctions in how the content of the action representation is processed (e.g., automatic vs. controlled), the amount of processing required (Dahm and Rieger, 2016), or differences in representational content (Frank et al., 2014; Schack et al., 2014). It is likely that experienced actions are represented more sparsely (i.e., the strong activation of a small neural population; Desimone, 1996) which may contribute to reduced performance times (due to neural efficiency), or reduced levels of necessary attentional control (due to increased automaticity of processes; see, Debarnot et al., 2014). This is an area that requires further empirical investigation to decipher the precise mechanisms contributing to or underlying experience effects in MI.

## Concluding Comments on Support for Simulation Theory

The preceding neural, neuropsychological, and behavioral findings converge to suggest that executed actions and cognitive motor states such as MI have a close correspondence. Specifically, both movement types appear to rely on similar mental representations that are embedded in neural motor systems, and both seem to have a causal role in generating actions through their activation of the motor system. MST offers a mechanism (i.e., simulation) for the functioning of action representations (e.g., MI), thereby relating cognitive motor states, such as MI, to actual action. MI has oftentimes been viewed as empirically intractable due its covert nature. However, neural-level investigation has brought imagery research into the realm of tangible, observable knowledge (e.g., see MacKisack et al., 2016). Further, such investigations have markedly increased over the past 15 years (see Di Rienzo et al., 2016). However, we propose that without understanding the cognitive organization and precise operational mechanisms of MI, any attempt to map its functional subcomponents in the brain is likely to be premature (but see Poldrack and Yarkoni, 2016, for some interesting new ideas about how informatics-based approaches can facilitate the delineation of brain-cognition mappings). Mapping is most meaningful if those subcomponents have been empirically associated with MI processes (and are not merely epiphenomenal; for further discussion on the contribution of neural-level analysis for the advancement of cognitive theory, see Coltheart, 2013; Mather et al., 2013). Further, as stated earlier, the discovery of common active brain regions during MI and ME does not arbitrate empirically between the explanations of MI offered by MST, emulation theory and grounded theory (Grush, 2004; Barsalou, 2008). We propose that in order to fully understand MI, and its underlying mechanisms, we must understand the cognition that supports it (which incidentally may also shed light on the precise mechanisms involved in actual motor control; see de Lange et al., 2008; Verbruggen et al., 2014).

## Does MST Provide an Adequate Understanding of the Cognitive Mechanisms Underlying MI?

The simulation mechanism alleged to operate during MI (which is charged with the task of rehearsing the neural motor system) has been linked to positive behavioral-level effects, such as enhanced action performance (for review, see Di Rienzo et al., 2016) in athletes (e.g., for reviews, see Driskell et al., 1994; Weinberg, 2008), medicine and rehabilitation (e.g., for review, see Page, 2000; Malouin and Richards, 2010; Arora et al., 2011; Schuster et al., 2011; but also see Braun et al., 2013), musicians (e.g., Ross, 1985; Theiler and Lippman, 1995; Bernardi et al., 2013) and dancers (Bolles and Chatfield, 2009; Girón et al., 2012), and modifications of functional connectivity in the motor system (Pascual-Leone et al., 1995; Lafleur et al., 2002; Jackson et al., 2003; Zhang et al., 2014). Additionally, impairments in the mechanism may underlie imagery-related psychopathology (e.g., delusions of control, see e.g., Jeannerod, 2001; PTSD and social anxiety disorder, see e.g., Moran et al., 2015). MST claims that MI, via simulation of action representations, “shapes the motor system in anticipation to execution” and this facilitates the subsequent actual action (Jeannerod, 2001, p. S103). Such facilitation is proposed as an explanation for improved movement performance following MI/MP. However, in this section, we question the extent to which MST is adequate for the task of explaining exactly how MI works.

Earlier in the paper, we explained MST’s proposition that imagined actions are actions, except that they are not executed. Expanding this idea, Jeannerod claimed that “everything that is involved in an overt action (i.e., the action goal, plan, motor program, and action consequences), except for the muscular contractions” (Jeannerod, 2004, p. 379) and their associated generated reafferent signals is also contained in the motor representations/images of MI (Jeannerod, 2001, 2004, 2006a). By implication, imagining an action is assumed to rely on the same mechanisms as those involved in actually executing it – except that overt movement is inhibited (Jeannerod, 2004, 2006a). If this proposition is valid, then firstly, the cognitive systems supporting ME should also be largely involved during MI, and secondly, any effects observed during voluntary movement should be observed during MI. Further, and perhaps the greatest implication of the claim of similar representations between ME and MI in MST, is that in order to maintain its covert status, MI must employ inhibitory mechanisms.

From the preceding analysis, it seems that MST relies on a rather ill-defined mechanism – simulation – that is held to operate during MI to plan, program, monitor, control and inhibit action, without explaining precisely how it does so, other than operating the neural motor system. Unfortunately, researchers applying the MST framework often seek to support the simulation hypothesis without questioning the nature of the mechanism itself or the processes that support it. In order to rectify this problem, we need to investigate how simulation functions, and whether or not it fully accounts for how MI works. We also need to identify the set of mechanisms purportedly simulated. In addressing these issues from Jeannerod’s theoretical perspective, we propose that the simulation process during MI serves at least three functions: (i) the selection and assembly of action elements stored in long term memory (LTM; a memory system whereby information that cannot be held in immediate, working memory is stored and exists apart from immediate cognitive processing; Jeannerod, 1994, 1997, 2006a; Jeneson and Squire, 2012); (ii) the monitoring of action simulation activity toward the goal (Jeannerod, 1994, 1997, 2006a); and (iii) the inhibition of overt movement (Jeannerod, 2001, 2004, 2006a).

Regarding the selection and assembly of action elements, according to MST, simulation does not merely signify the reactivation of previously executed actions (held in LTM), but instead is purported to activate mechanisms that select and assemble “unspecific elements” of action or schemas during action planning (Jeannerod, 2006a, p. 134). Jeannerod (1997, 2006a) suggested that a potential mechanism may be somewhat like Norman and Shallice’s (1986) ‘supervisory attentional system’ (SAS) – a control mechanism for biasing selection processes. Attention is a multifaceted process that incorporates both inhibitory and facilitatory mechanisms during information processing, for actively suppressing distractor information and enhancing relevant information, respectively (Tipper, 1985; Kok, 1999). The construct is central to cognitive neuroscience because it explores the mechanisms by which “voluntary control and subjective experience arise from and regulate our behavior” (Posner and Rothbart, 2007, p. 1). However, in MST, potential attentional mechanisms (i.e., SAS) are insufficiently examined in relation to action representation (see Jeannerod, 1997, 2004, 2006a), and nowhere can we find it directly related to MI. Therefore, the precise role of attentional mechanisms in MI and the issue of how they relate to simulation are curiously unclear in MST. It may be that as simulation is considered to “rehearse the short-term, fast and automatic unfolding of movement” (Jeannerod, 2006a, p. 140) that the SAS operates in accordance with the action goal, to actively bias the activation and inhibition of competing elements and thus control appropriate action selection. However, it remains unclear as to whether such an attentional mechanism is solely responsible during MI for directing attention to action planning operations or is responsible for other processes – such as action initiation, direction, monitoring, adjusting, completion, completion verification, or even for the conscious awareness of (imagined) kinesthetic sensations typically experienced during MI. It can be noted at this stage that, according to MST, imagined actions are assembled from unspecific movement elements, and therefore although this should facilitate novel action learning using MI, evidence exists to the contrary – learning does not occur without prior physical movement experience (Mulder et al., 2004; Olsson and Nyberg, 2010).

According to MST, motor representations/images are formed prior to execution (recall the representation-execution continuum), and they include the action goal and anticipated action effects – anticipated through simulation (Jeannerod, 2006a). Research demonstrates that during actual action attention modulates (Jones et al., 2013) or enhances the precision of action-effect prediction and intensifies sensory information (Kok et al., 2012). Further, attention itself can act as an anticipatory mechanism (e.g., see, Jones et al., 2013). Therefore, it is possible that during MI, simulation relies on attentional mechanisms for predictive processing. To test this possibility, empirical studies exploring interactions between attention and MI would be valuable, and would also help to clarify the role of attention in simulation processes. This line of investigation could be particularly fruitful given that a typical effect of voluntary (actual) action is sensory attenuation (i.e., where sensory signals are usually attenuated during predicted actions; see, e.g., Blakemore et al., 1998; Roussel et al., 2013) rather than intensified sensory information (which can arise from attention to action-effect prediction; Kok et al., 2012). So, an examination of whether or not the effects observed during voluntary actual action (e.g., sensory attenuation) are present in MI might help to elucidate the neurocognitive mechanisms underlying MI. If, for example, sensory attenuation effects *are* also present in MI, this would strengthen the functional equivalence hypothesis of simulation theory. Demonstrating similar effects in both ME and MI would support the idea of shared representations and mechanisms, but might suggest that sensory information is also simulated. Consequently, the current MST framework would need to be extended to account for this possibility, perhaps borrowing from emulation theory which promotes the idea that both motor and sensory systems are simulated (Grush, 2004).

Interestingly, research has raised the possibility that “attentional effort” (or the motivated allocation of attentional resources to satisfy cognitively challenging demands; Kahneman, 1973), and its associated physiological component of arousal, may mediate MI processes. For example, studies have demonstrated that temporal congruence between actual and imagined movement performance durations is closest when participants are in an aroused rather than relaxed state (Louis et al., 2011), and that the greatest disparities are found when tasks are performed early or late in the day (Gueugneau et al., 2009) – times when levels of circadian arousal are lowest (van der Heijden et al., 2010). As yet, however, the extent of attentional effort involved during MI in comparison to ME has not been investigated. In the absence of studies on this latter issue, we cannot discount the possibility that the demonstrated temporal incongruence between MI and ME at times of low arousal levels are due to altered recall, rather than due to attentional processes. In this regard, the neurochemical norepinephrine associated with arousal levels can mediate memory retrieval processes (see, e.g., Murchison et al., 2004). This might provide support for a grounded theoretical conceptualization of simulation, whereby simulation involves the re-enactment of multi-modal representations from memory. In this case, modulated memory processes might mediate MI ability.

As previously mentioned, the second proposed function of the simulation process during MI is to monitor the action simulation activity toward the goal. In the context of MST and ME, motor representations are considered to operate in a similar manner to the forward models of computational motor-control theory (e.g., Wolpert et al., 1995), in that, motor-signal copies (efferent copies) are used to predict the new sensory state (i.e., the action outcome) without actually performing the action (Jeannerod, 1994, 1997). This anticipated new state is encoded in the action goal representation and its persistent activation at the neural level provides an internal mechanism (i.e., without need for external sensory input) for comparing the unfolding action with the desired action (Jeannerod, 1997). The goal representation remains active until the desired new state is achieved. In relation to MI, MST posits that the role of simulation is “to ‘evaluate’ the feasibility of the action, its potential consequences and its adequacy with respect to the anticipated goal” (Jeannerod, 2006a, p. 165). However, MST does not specifically address the issue of whether or not a comparator mechanism operates during MI or simulation, for these evaluation processes – for assessing the adequacy of the action for achieving the desired goal. If during MI, as MST claims, the “goal is not reached even though all the conditions for potentially reaching it are fulfilled” (Jeannerod, 2004, p. 376), and thereby presumably remains activated, the question arises as to how imagined actions are terminated and the simulation mechanism thereby disengaged. This process may be the result of either comparator or attentional mechanisms (or both). Additionally, during MI each step of, for example, a sequential complex action is subjectively performed, and experienced as being completed, in relation to the desired action goal. Although MST claims that action consequences are predicted via simulation processes, and evidence suggests that awareness of the kinesthetic feeling of imagined movement comes from action-effect prediction (Tian and Poeppel, 2010), it is unclear as to whether subjective awareness is an adequate mechanism for simulation/imagined action termination. Emulation theory (Grush, 2004) offers a possible solution to this problem because it proposes that sensory feedback is also simulated. In this case, simulated sensory feedback would allow progress monitoring and control of the imagined action through comparison between simulated action and its simulated feedback in relation to the anticipatory representation of desired action effects (e.g., Pezzulo and Castelfranchi, 2009). Further, simulated feedback would also facilitate termination of the imagined action. Overall, empirical investigation is required to explore the degree to which MI requires comparator mechanisms for accurate functioning. If instead, MI employs anticipatory-associative simulations, as discussed earlier (Hesslow, 2002, 2012), comparison mechanisms may not be necessary as the simulation process would be fuelled by previously experienced and learned associations. However, even in this case, the problem arises as to how exactly the simulation process (i.e., MI) is terminated. Alternatively, if, as grounded theories suggest, imagined action performance re-enacts a previously learned action that is stored in memory, then, attentional processes that select and inhibit the appropriate motor aspect of the multimodal representation could oversee the initial re-enactment process. Then, once underway, the simulated action would automatically terminate according to the memory held. Unfortunately, the mechanisms that might explain how the simulation process is terminated have not been adequately addressed by empirical studies to date. Accordingly, research is urgently required to investigate this important question.

Inhibition of overt movement during MI is considered to be a function of the simulation process (as delineated earlier in the paper; Jeannerod, 2001, 2004, 2006a). During MI, inhibition is a key mechanism that differentiates imagery from actual execution, as it involves the complete suppression of overt movement behavior (Ridderinkhof et al., 2011). For cognitive researchers, inhibition may be defined as “the stopping or overriding of a mental process, in whole or in part, with or without intention” (MacLeod, 2007, p. 5). In this sense, and in relation to MST, inhibition during MI concerns the withholding of some degree of mental processing so that the probability of movement execution is reduced. The majority of studies investigating the inhibitory mechanisms operating during MI have focused on neural or neurocomputational substrates (see Ridderinkhof et al., 2011; Guillot et al., 2012b; Rieger et al., 2016). Literature in this domain has sought to decipher whether inhibitory processes are, firstly, integrated into MI processes so that active inhibition is not required (i.e., motor commands remain at a subthreshold level), or secondly, actively implemented during MI to either inhibit all movement (global inhibition), action-specific movement, or effector-specific movement (see Jeannerod, 2001; Guillot et al., 2012b; Rieger et al., 2016). Tentative conclusions from this literature suggest that inhibitory processes may be tightly coupled with MI processes to sustain a subthreshold level of motor command throughout imagined movement (e.g., Jeannerod, 2001; Stinear, 2010; Guillot et al., 2012b). At a cognitive level, this might imply that during MI, inhibitory processes are built in to the motor intention (see, Ridderinkhof et al., 2014), and have a role in guiding motor processes and preventing execution of the motor program. If inhibitory mechanisms operate at earlier intentional stages of MI it would be expected that a limb involved in an imagined movement would be unavailable for actual movement. Interestingly, Bach et al. (2014) discovered that participants found it more difficult to respond with a body-part (hand or foot) when that body-part was concurrently engaged in MI, and instead typically responded with another body part. The authors link this to motor-planning processes, involving goal and action response binding, that reduce the availability of the body-part for unrelated responses. These findings also support the idea that inhibitory processes are closely associated with intentional processes and further suggest that such inhibition is effector-specific (see also, Rieger et al., 2016). However, it should be noted that these findings might also be explained by appealing to attentional mechanisms. Specifically, it is possible that attentional resources were invested in the MI task, and that the structural interference arising from resource competition (i.e., competition for similar motor systems) prevented participants from successfully using the same effector for execution. Recall that MST claims that MI relies on the same mechanisms as ME – expect that overt movement is inhibited (Jeannerod, 2001, 2004, 2006a). In summary, despite increased research interest in this topic, little progress has been made in clarifying precisely *when* and *how* (e.g., through intentional or attentional mechanisms) inhibition is implemented during MI.

Given this impasse, we propose that some insight into inhibitory mechanisms might be gained from the study of gestures during speech. According to Hostetter and Alibali (2008), gestures arise from a combination of language processing and MI. Specifically, within these researchers’ ‘gestures as simulated action’ framework, when speakers talk about actions, they simultaneously engage in simulation or MI of those actions. Gestures arise if the motor simulations are not inhibited. Failure to inhibit MI processes is thought to result from strong neural activation of the motor representations which arises from action experience (Sassenberg and van der Meer, 2010; Hostetter, 2014). Further, gestures are more likely to be produced when speaking a word that has strong action characteristics (Hostetter, 2014). This finding is consistent with literature that demonstrates the involvement of the motor system during action-related language (Pulvermüller et al., 2005; Holt and Beilock, 2006; Hauk et al., 2008; Andres et al., 2015). Interestingly, gesture research is also compatible with the claims of grounded theories that an action word may trigger its multi-modal representation which is simulated to extract meaning. Gestures would result from a failure to inhibit motor simulation of the representation. By contrast, MST cannot easily account for this semantic-pragmatic interaction during speech gestures of action words as it claims that simulations during MI are in a pragmatic format (a ‘how to do’ format; Jeannerod, 2006a). Within the MST perspective, the word would activate motor systems after its meaning has been extracted which would elicit a motor image (Jeannerod, 2006a). Overall, the nature of inhibition in MI is an under-investigated area and further research is needed to investigate precisely when and how inhibitory processes are engaged and controlled during MI.

## New Directions for MI Research

Throughout this paper we have argued that although MI is a psychological process, its psychological bases remain surprisingly unclear. Given the existing imprecise determination of mechanisms of MST, and indeed of other accounts of simulation, and the poorly understood psychological mechanisms underlying MI, it seems timely to re-evaluate approaches to investigating how and under what conditions MI operates. Recent ideas relating to grounded theoretical approaches to the intertwined development of higher cognitive and executive functions and motor control (Pezzulo and Castelfranchi, 2009; Gottwald et al., 2016) provide promising directions for MI research.

Before we conclude this section, it may be fruitful to consider a topic which may enable us to arbitrate empirically between contrasting predictions from grounded theory and MST. One such topic is the learning and performance of novel actions. Grounded theory predicts that performance of novel actions through MI would not be possible as simulation is regarded as a mechanism that re-enacts multimodal representations held in memory (Barsalou, 2008). Consequently, if an appropriate representation does not exist in memory for a desired movement, it cannot be simulated, or by association, imagined. Conversely, regarding MST, learning novel movements through MI appears quite tenable, as it is claimed that simulation mechanisms allow imagined action performance by assembling motor representational elements anew, rather than utilizing complete action representations that are stored in memory (Jeannerod, 2006a). Some evidence exists that MI positively affects motor skill learning, for example, after only a brief period of physical practice – necessary for familiarization and pretesting – (Gentili et al., 2006, 2010), and even without prior physical practice (Pascual-Leone et al., 1995; Kraeutner et al., 2016). However, there is also contradictory evidence that motor learning does not occur via MI unless there is prior movement experience (Mulder et al., 2004; Olsson and Nyberg, 2010). Predictive processing views of MI maintain that motor acquisition through MI is linked to the extent of experience an individual has with the particular action (see Ridderinkhof and Brass, 2015). However, it should be noted that studies have found changes in brain activity and functional connectivity following MI/MP (see Pascual-Leone et al., 1995; Lafleur et al., 2002; Jackson et al., 2003; Debarnot et al., 2014; Zhang et al., 2014). Changes in brain activity following MI might reflect the benefit of simulation processes in shaping the motor system (action anticipation) to facilitate subsequent action execution (MST), or in refining the representational re-enactment process, whereby simulations are only performed for those components of the representation that are essentially required for action (grounded theory).

## Concluding Comments

Over the past 15 to 20 years, MST has been consistently invoked in an effort to explain how MI works (e.g., Holmes and Collins, 2001; Erlacher and Schredi, 2008; Munzert et al., 2009; Munzert et al., 2015; Gentsch et al., 2016). But does this theoretical approach provide an adequate theoretical account of the cognitive mechanisms underlying MI? In an effort to answer this question, the present article had two objectives. Firstly, it attempted to clarify the terminology of MST, summarize its key postulates and to evaluate available evidence cited in support of them. In addition, it sought to evaluate the degree to which MST provides a sufficient explanation of the cognitive mechanisms underlying MI. To this end, key anatomical brain regions associated with actual and imagined movement were highlighted, and in support of simulation theory, considerable regional overlap was identified. However, subtle differences in the neural-networks underlying MI and ME were evident and emphasize the importance of understanding MI at different levels of analysis. Overall, we have argued that MST relies on an underspecified mechanism – simulation – as the operational characteristics of the mechanism itself are unclear. Thus, empirical inquiry and cognitive-level process models that explain precisely how the simulation mechanism operates during MI are required to explain, for example, how simulation is initiated, continuously generated, and terminated, or if it is under constant conscious control. Further, while MST claims that ME and MI rely on the same set of mechanisms, few of these are precisely delineated. Based on Jeannerod’s work, we have suggested that the simulation process during MI may operate at least three further mechanisms, namely, attentional mechanisms for the selection and assembly of motor elements, comparator mechanisms for the monitoring of imagined action toward a goal, and inhibitory mechanisms for preventing overt execution during MI. It is apparent from the research discussed that the psychological architecture of MI remains poorly understood. Without fully understanding the cognition underlying MI, other theories emphasizing the reliance of cognition on sensorimotor activity, such as grounded theory, or emphasizing simulated sensory feedback, as in emulation theory, cannot be discounted as more adequate explanations for how MI works. In this regard, we suggest that an integration of cognitive, neural and behavioral level research will likely be most productive in providing comprehensive knowledge of exactly how MI functions.

## Author Contributions

All authors listed, have made substantial, direct and intellectual contribution to the work, and approved it for publication.

## Conflict of Interest Statement

The authors declare that the research was conducted in the absence of any commercial or financial relationships that could be construed as a potential conflict of interest.
